# *Atriplex mollis* Desf. Aerial Parts: Extraction Procedures, Secondary Metabolites and Color Analysis

**DOI:** 10.3390/molecules23081962

**Published:** 2018-08-06

**Authors:** Nassima Boutaoui, Lahcene Zaiter, Fadila Benayache, Samir Benayache, Francesco Cacciagrano, Stefania Cesa, Daniela Secci, Simone Carradori, Anna Maria Giusti, Cristina Campestre, Luigi Menghini, Marcello Locatelli

**Affiliations:** 1 Unité de Recherche Valorisation des Ressources Naturelles, Molécules Bioactives et Analyses Physicochimiques et Biologiques, Université Frères Mentouri, Constantine 1, Route d’Aïn El Bey, 25000 Constantine, Algeria; boutaoui.nassima@gmail.com (N.B.); lahcene.zaiter@yahoo.fr (L.Z.); fbenayache@yahoo.fr (F.B.); sbenayache@yahoo.com (S.B.); 2 Department of Pharmacy, University “G. d’Annunzio” of Chieti-Pescara, Via dei Vestini 31, 66100 Chieti, Italy; francesco.cacciagrano@studenti.unich.it (F.C.); simone.carradori@unich.it (S.C.); cristina.campestre@unich.it (C.C.); luigi.menghini@unich.it (L.M.); 3 Department of Drug Chemistry and Technologies, Sapienza University of Rome, P.le Aldo Moro 5, 00185 Rome, Italy; stefania.cesa@uniroma1.it (S.C.); daniela.secci@uniroma1.it (D.S.); 4 Department of Experimental Medicine, Sapienza University of Rome, P.le Aldo Moro 5, 00185 Rome, Italy; annamaria.giusti@uniroma1.it

**Keywords:** *Atriplex mollis*, SFE, MAE, HPLC-PDA, color analysis, pigments

## Abstract

A method using high-performance liquid chromatography coupled with a photodiode array detector was proposed for the rapid characterization of different phenolic constituents from the extracts of *Atriplex mollis* aerial parts. *Atriplex* species are known for their multiple biological activities, but no information is available in the literature about *A. mollis*. With the aim to firstly characterize the main secondary metabolites of this plant, so as to orient better the biological evaluation, we applied three different extraction procedures and compared the chromatographic results. Microwave-assisted extraction gave the best yield and recovery of important compounds such as gallic acid, catechin, chlorogenic acid, *p*-OH benzoic acid, rutin, sinapinic acid, *t*-ferulic acid, naringenin and benzoic acid. These constituents belong to three important chemical classes: phenolic acids, flavonoids and monoterpenes. Color evaluation and analysis of chlorophylls (a and b) and carotenoids complete the preliminary profile of this plant. From these analyses, *Atriplex mollis* is a source of bioactive compounds (especially rutin, *t*-ferulic acid and gallic acid) and could be recommended as a plant of phyto-pharmaceutical relevance, opening new perspectives on this salt-tolerant plant.

## 1. Introduction

Chenopodiaceae are widespread throughout the world, but have a strong preference for saline clay soils, living mainly in arid and semi-arid climates as xero-halophyte saltbushes [1]. Over 400 species of *Atriplex* have been identified. The Mediterranean basin has 40–50 *Atriplex* species with 15 growing in Algeria [2,3]. Chenopodiaceae are dicotyledonous flowering plants with no petals, the leaves are often covered by trichomes, pedicellate glands with heads formed by a large cell filled with salty water. The stem cross section shows vascular bundles widely scattered on stele or distributed in multiple concentric rings, as adaptive arrangement to salty and sandy environment [4,5]. Unlike other vascular plants, salt-tolerant species, endowed with high physiological plasticity, are able to survive under stressful conditions. Indeed, as halophytic adaptation to the salinity of soils, these species raise their osmotic concentration to high values as they consequently accumulate a large quantity of salts [6,7,8].

The previous phytochemical analyses of some *Atriplex* spp. reported the presence of several classes of secondary metabolites such as saponins, glycosides, flavonoids, tannins, terpenoids, alkaloids, and proteins as well as amino acids and long-chain alcohols. Additionally, no essential oils were found in these species, but they were characterized by high amounts of sulfur-derived compounds and minerals. Particularly, the content of proteins and amino acids changed during plant growth [9,10]. Compared with other species, and as far as our literature survey could reveal, no phytochemical investigations on *Atriplex mollis* Desf. aerial parts were previously reported. This prompted us to choose this species (widespread and endemic in Algeria) for an extensive phytochemical study with the aim of determining the main bioactive secondary metabolites (phenolics, carotenoids, and chlorophylls). Indeed, halophytes are well known for their ability to react with toxic reactive oxygen species, since they evolved an over-expressed antioxidant system and, as a response to abiotic stress, provide high levels of phenolic compounds [9]. Additionally, it was demonstrated that specific responses to salt stress adaptation in halophytic plants could be related to flavonoids, sulfolipids, and polyphenols over-production [11,12].

In order to contrast the salt-induced damage, halophytes also increase the chlorophyll and carotenoid content for the maintenance of cellular and physiological homeostasis, thus confirming the important role (and, consequently, the determination) of these small non-enzymatic molecules.

Natural polyphenols (anthocyanins, tannins, flavanones, isoflavones, resveratrol and ellagic acid) are currently used in the industry as nutraceuticals and/or functional foods. Among them, halophytes could be valuable sources for economical applications not only for their phenolics pattern, but also for their carotenoids and pigments content. They are important in plants for normal growth development and defense. Furthermore, due to the presence of these compounds, this plant could be popularized for food and medicinal purposes as antioxidant, anti-allergic, anti-inflammatory, anti-thrombotic, cardio-protective and vasodilatory agent.

Ethnobotanists have not suggested any traditional use of this species, although some biological activities have already been described for various species of Chenopodiaceae [9]. The majority of studies on *Atriplex* spp. investigated adaptive morphological and physiological responses under salinity stress [13] and the potential use as a fodder for livestock [14], without considering this native Algerian species as a suitable candidate to be used as a cash crop (edible and ornamental plant, fodder, bio-fuel, folk medicine, and source of chemicals) or investigating the chemical composition. In recent years, the polyphenol market has increased attention on nutraceutical and functional foods for human consumption, and prompted research towards the development of extractive technologies to accomplish the requirements of food industry.

In the framework of green chemistry, the present study was aimed at screening and identifying for the first time the main components from different extracts (maceration, supercritical carbon dioxide extraction, and microwave-assisted extraction) of *A. mollis* aerial parts monitored by a validated multi-component analysis with high performance liquid chromatography coupled with photodiode array detector (HPLC-PDA). As recently highlighted in the literature, due to the huge differences in terms of polarity, solubility, stability, and concentration of these metabolites, a single solvent or extraction procedure may not furnish a comprehensive total metabolite pattern. The precise knowledge of the specific metabolite content should guide a rational biological evaluation according to the concept of a “hypothesis-driven approach”, thus preserving the limited biodiversity of this endemic species.

## 2. Experimental Section

### 2.1. Chemicals

Standard chemicals for the wide chromatographic analyses (caffeic acid, gallic acid, naringenin, carvacrol, catechin, chlorogenic acid, epicatechin, 4-hydroxy-benzoic acid, *t*-ferulic acid, naringin, vanillic acid, *p*-coumaric acid, rutin, syringic acid, benzoic acid, *o*-coumaric acid, 3-hydroxy-benzoic acid, sinapinic acid, 2,3-dimethoxy-benzoic acid, quercetin, harpagoside, *t*-cinnamic acid (all purity > 98%)) as well as HPLC-grade methanol, chloroform, *n*-butanol, ethyl acetate, acetonitrile and glacial acetic acid were purchased by Sigma-Aldrich (Milan, Italy) and required no further purification. Double distilled water was produced by a Millipore Milli-Q Plus device (Millipore Bedford Corp., Bedford, MA, USA). All extractions were preliminarily monitored by thin layer chromatography on 0.2 mm thick silica gel plates (60 F_254_ Merck, Kenilworth, NJ, USA) and the spots were visualized at 254 and 365 nm under an ultraviolet (UV) lamp. Flash column chromatography was performed using Biotage Isolera™ system with cartridges packed with Biotage^®^ HP-Sphere™ spherical silica (Biotage AB, Uppsala, Sweden).

### 2.2. Plant Material

The plant material was collected from Bershka region (Coordinates: 34°48’N 5°41’E), Algeria, in May 2016. The plant was identified by Prof. M. Kaabeche (Biology Department, University of Setif 1, Algeria). A relative specimen voucher has been registered in the Herbarium of Frères Mentouri University, Algeria.

## 3. Extraction Techniques

### 3.1. Maceration and Further Fractionation

A proper quantity (2 kg) of aerial parts of *A. mollis* was dried at room temperature (until constant weight, 842 g of dry weight), cut and powdered (50 mesh), and macerated three times (24 h for each time with fresh solvent mixture) with ethanol:water (80:20, *v*:*v*, 15 L). After filtration and evaporation at T < 40 °C, the crude extract was partitioned between water and solvents at increasing polarity (following a liquid–liquid extraction) in order to obtain more metabolite-oriented subfractions: chloroform, ethyl acetate and *n*-butanol. Each extract was dried with anhydrous Na_2_SO_4_, then filtered and evaporated under reduced pressure at T < 40 °C to give CHCl_3_ extract (2.57 g), EtOAc extract (2.67 g) and *n*-BuOH extract (20.31 g). 

**Figure 1:**
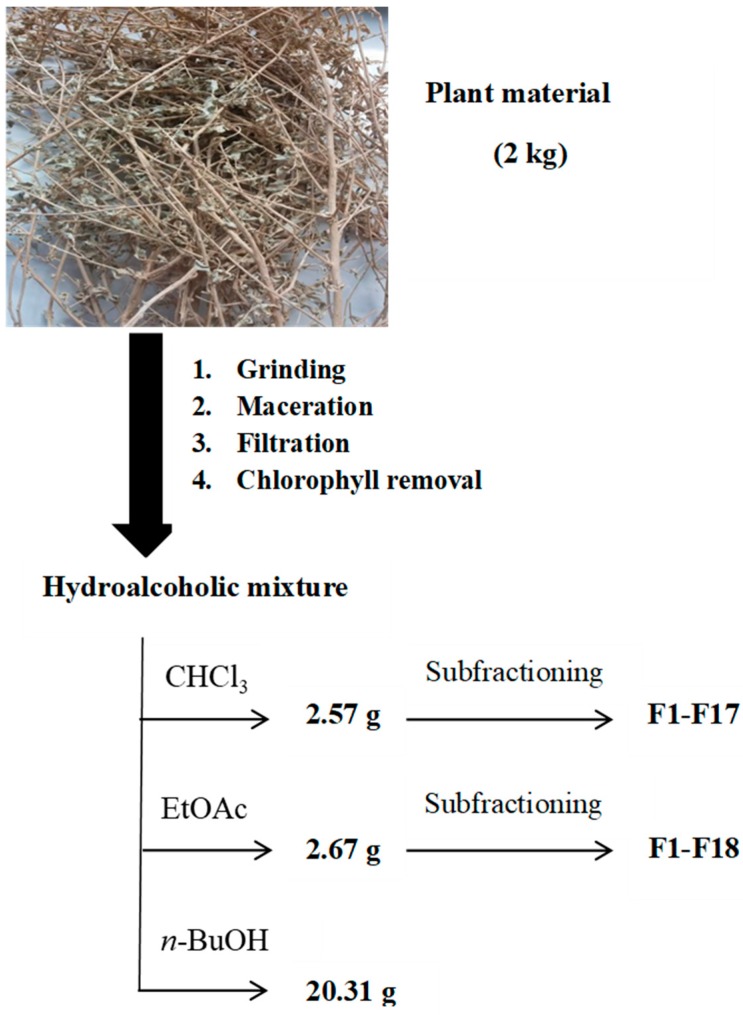
Experimental maceration and subfractioning flowchart performed on *A. mollis* aerial parts.

The chloroform extract was further fractionated by flash chromatography (MeOH/CHCl_3_, step gradients) to provide 17 fractions. Successively, the EtOAc extract was fractionated by flash chromatography (MeOH/CHCl_3_, step gradients) to provide 18 fractions (Figure 1). Each sample was stored in a sealed bag in the dark at −20 °C.

### 3.2. Supercritical Fluid Extraction (SFE)

The extractor for Supercritical fluid extraction (SFE) was equipped with a carbon dioxide delivery pump (PU-2080-CO_2_, Jasco, Tokyo, Japan) connected to a thermostatic chamber with a 50 mL extraction column, an UV-Vis detector with high pressure cell (875-UV, Jasco) and an automatic back pressure regulator (BP-2080 plus, Jasco). 20 g of powdered plant material (50 mesh) were packed in a 50 mL SFE extraction bulk and then exposed to a dynamic extraction with a CO_2_ flow-rate of 3 mL/min (40 °C, 60 min). Two discrete pressure conditions, namely 10 and 30 MPa (carbon dioxide density: from 628.7 to 910 kg/m^3^), were chosen in order to tune supercritical fluid density. After vacuum filtration over Chromafil^®^ PET 20/25 (0.2 µm pore size, Machery-Nagel AG, Oensingen, Switzerland) into dark glass vials, the extracts were pooled at room temperature and kept under refrigerated condition until chromatographic analyses. 

### 3.3. Microwave-Assisted Extraction (MAE)

Microwave-assisted extraction (MAE) was carried out by means of a single-mode microwave reactor automatic Biotage Initiator^TM^ 2.0 (Biotage AB, Uppsala, Sweden) characterized by power range 0–300 W and 2.45 GHz microwaves. The internal vessel temperature was monitored by an infrared (IR) sensor probe. Powdered samples (50 mesh) were suspended in water to obtain a liquid-to-solid ratio of 20:1 (*v*:*w*) and transferred into a sealed vial. MAE was performed at discrete temperatures (40, 60, 80, 100 or 120 °C) for 5, 10 or 15 min and then cooling by pressurized air. For many solvents, the superboiling temperature under microwave irradiation, in a closed vessel, can be 10–40 °C higher than their classical boiling points [15]. Then, the suspension was vacuum filtered over Chromafil^®^ PET 20/25 (0.2 µm pore size, Machery-Nagel AG, Oensingen, Switzerland) into dark glass vials and the extraction solvent (water) was freeze-dried by lyophilisation (VirTis, Gardiner, NY, USA). The dried samples were carefully packed in an airtight polyethylene bag at −20 °C until further chromatographic analyses.

### 3.4. HPLC-PDA Method for the Analysis of Phenolic Compounds

HPLC-PDA phenolic determination followed a published and validated method [16]. All dried extracts were weighted, dissolved in mobile phase and injected (20 µL) into the HPLC-PDA system. Data are displayed as mean ± standard deviation (SD) of three independent measurements. The identification of each phenolic compound was assessed considering UV-Vis spectra and retention time compared to pure standard compounds. The quantification was carried out using an external standard method with commercially available and high purity phenolics. Analytical figures of merit on the validation of this method were corroborated after evaluation of matrix interferences. Thus, this method was reasonably sensitive to detect the compounds in real matrices.

## 4. Color Evaluation

Colorimetric CIELAB parameters, namely L*, a*, b*, C*_ab_ and *h*_ab_, are defined by the “Commission Internationale de l’Eclairage” and determined on the ground *A. mollis* aerial parts using a colorimeter X-Rite SP-62 (X-Rite Europe GmbH, Regensdorf, Switzerland), characterized by a D65 illuminant and an observer angle of 10°. Three color parameters were assessed: L* (lightness), a*, greenness (−a*) or redness (+a*) and b*, blueness (−b*) or yellowness (+b*). Cylindrical coordinates C*_ab_ and *h*_ab_ were extrapolated from a* and b* following the equations C*_ab_ = (a*^2^ + b*^2^)^½^ and *h*_ab_ = tan^−1^(b*/a*) [17]. The dried aerial parts of the plant were firstly powdered (50 mesh) in a commercial blender (MJ-220BP01A, Guangdong Co., Ltd., Guangdong, China) for homogeneity. The obtained powders were divided into four aliquots with a little different apparent granulometry and analyzed. The results are given as mean value ± standard deviation (SD).

## 5. Pigment Determination

The total carotenoids and chlorophylls a and b determination for the *Atriplex* sample was carried out according to Solovchenko and co-workers (2001) with slight modifications [18,19]. The sample was powdered (50 mesh) with mortar and pestle in 5 mL of chloroform-methanol (2:1, *v*/*v*) mixture containing 0.01% BHT to limit peroxidation. In addition, homogenization was performed with MgO (50 mg) to inhibit chlorophyll pheophytinization. After filtration, distilled water was added in order to reach the 20% of the total extract volume. Lastly, the suspension was centrifuged for 18 min at 3000× *g* at 10 °C to achieve the phase separation. Absorption spectrum of the chloroform phase was registered in the range of 350–800 nm with a Beckman Coulter DU 800 instrument with a spectral resolution of 0.5 nm at 20 °C. Both chlorophyll and the total carotenoid amounts were evaluated according to Wellburn (1994) [20]. Data are displayed as mg/g dry weight (DW) ± SD (n = 3).

## 6. Results and Discussion

### 6.1. HPLC Analysis of Hydroalcoholic Extract and Subfractions

In order to improve the extraction methods and the yield of the different secondary metabolites extracted by medicinal plants, we have carried out this analysis in which the polyphenols of the different hydroalcoholic extracts and their isolated fractions could be qualified and quantified (µg/g DW ± SD). In Table 1, we reported only the subfractions containing at least one of the phenolic compounds detectable by our chromatographic procedure.

Extracts at increasing polarity (chloroform, ethyl acetate and *n*-butanol) were obtained from the ground aerial parts of *A. mollis* and analyzed by our simple, accurate (precise and true) HPLC-PDA method. These constituents belong to three important chemical classes: phenolic acids, monoterpenes and flavonoids. The ethyl acetate extract was the most characterized in phenolic compounds by comparison with the other two extracts. It was characterized by high amounts of *p*-hydroxy benzoic acid, rutin and catechin with the highest concentration in some isolated fractions (115 µg/g, 65 µg/g, and 34 µg/g, respectively). It is also worth noting that epicatechin, 3-OH,4-MeO benzoic acid and benzoic acid were identified in three subfractions of ethyl acetate extract at a lower extent. Sixteen compounds were detected in the fractions isolated in the chloroform extract, with epicatechin, *p*-coumaric acid and vanillic acid at the highest concentration (158 µg/g, 24.9 µg/g, and 42.2 µg/g, respectively). Carvacrol was also identified in the chloroform extract with the concentration of 0.44 µg/g in the fraction F7. Finally, eight compounds were assessed in the *n*-butanol (*n*-BuOH) extract, which proved to be the less enriched in phenolic compounds, among them, the flavonoid rutin (12.3 µg/g), the phenolic acid 3-hydroxy benzoic acid (1.5 µg/g) and the monoterpene carvacrol (0.15 µg/g). Collectively, a specific phenolic profile with predominant amounts of rutin, 3-hydroxy benzoic acid and chlorogenic acid was evidenced by these analyses.

### 6.2. HPLC Analysis of Supercritical Carbon Dioxide Extracts

In order to compare qualitative and quantitative results from different extraction methods, we also performed supercritical fluid extraction. The SFE-derived extract quality was evaluated in terms of dry extract yield and recovery of secondary metabolites from aerial parts of *A. mollis*. The SFE was carried out at fixed temperature (40 °C) to avoid phenolic degradation/oxidation and at two discrete pressure values (10 and 30 MPa). The better extraction, expressed as yield (yield 0.62% for 10 MPa and 0.82% for 30 MPa) and quantity of secondary metabolites, resulted at higher pressure (Table 2). Twelve and eleven metabolites were identified and quantified in the extracts at 10 and 30 MPa, respectively. These constituents belong to the chemical classes of phenolic acids, flavonoids and monoterpenes. Phenolic acids identified were gallic acid, vanillic acid, syringic acid, *p*-coumaric acid, sinapinic acid, *t*-ferulic acid, 2,3-diMeO benzoic acid, and benzoic acid, with sinapinic acid resulting in the highest concentration (2.39 µg/g). Conversely, flavonoids were identified as catechin, epicatechin, rutin with epicatechin resulting the most abundant (0.76 µg/g). Additionally, in the 30 MPa extract an iridoid glycoside was identified—harpagoside—at concentration of 0.20 µg/g. Several investigations have found that harpagoside-containing extracts have good anti-inflammatory and analgesic activities in multiple models of acute inflammation [21,22]. Collectively, SFE provided a relatively small amount of phenolic compounds due to the non-polar nature of the solvent.

### 6.3. HPLC Analysis of Microwave-Assisted Extracts

Lastly, keeping constant the use of a non-toxic solvent, we performed MAE to study the recovery of selected metabolites from *A. mollis* aerial parts. We carried out the extraction with water as polar solvent (solvent volume = 20 mL, material amount 1 g, extraction time = 10 min) to evaluate the best extraction temperature ranging from 40–120 °C [15]. We observed that the best extraction yield was obtained at 80 °C for 10 min (yields between 8.3–10.1% of DW). Shortening (5 min) or prolonging (15 min) the extraction time at 80 °C did not significantly improve the total phenolic yield.

Twelve compounds were identified and are listed in Table 3. As can be seen, the analyzed extract comprehended a large number of compounds belonging to phenolic acid and flavonoid classes. Among these flavonoids, rutin was identified to be the most abundant (486 µg/g, as expected using water as the solvent), while among phenolic acids *p*-OH benzoic, vanillic, *p*-coumaric, sinapinic, and *t*-ferulic acids were identified, with vanillic acid and *t*-ferulic acid having the highest concentration (125 µg/g and 95.5 µg/g, respectively).

Despite the fact that gallic acid, *t*-ferulic acid and rutin could be also recovered better at higher temperature (100 °C), up to 80 °C other compounds were detected in a considerable quantity such as catechin, *p*-OH benzoic acid, vanillic acid, *p*-coumaric acid and sinapinic acid. Naringenin and catechin were found only at lower temperature (40 °C), whereas benzoic acid could be extracted only at higher temperature (120 °C) [15]. Collectively, most of these phenolic compounds were detected in other *Atriplex* spp. (e.g., aerial parts of *A. halimus*), but a direct qualitative comparison is difficult because of the different extraction conditions reported in the literature [23,24,25,26].

The role of secondary plant metabolites such as phenolic acids and flavonoids in the prevention of human diseases has been widely investigated. In detail, vanillic acid (4-hydroxy-3-methoxybenzoic acid) was shown to display an interesting pharmacological activity. Experimental studies have provided evidence of effectiveness on cardiovascular [27], gastrointestinal [28] and liver diseases [29] by means of inhibition of inflammation markers synthesis and release. Among flavonoids, rutin was also shown to exert antioxidant, anti-inflammatory, hepatoprotective, nephroprotective, and neuroprotective activities [30,31,32,33]. 

### 6.4. Color Analysis

Dried aerial parts of *A. mollis* were submitted to colorimetric evaluation adopting CIELAB coordinates, which are directly useful for color evaluation by human vision. In this work, the aim of the reflectance colorimetry was limited to givg a first characterization of a new matrix. For this reason, the aerial parts of the plant, only powdered, were submitted to this fast and cheap analysis. The samples showed non-homogeneous coloring, in which at least three different color shadings could be observed. There was a light brown color typical of ligneous material, a clear gray-green or beige in larger parts and a lighter green in thinner parts. The mortar-homogenized samples did not show the brown color anymore, but a more brilliant green color was accompanied by orange shades corresponding to slightly higher L* values (66 vs. 62) and to a slight increase of the reflectance curves. The mean value ± standard deviation is reported in Table 4. The relatively high value of b* is probably related to the carotenoid content (see paragraph below), which entirely accounts for the color purity expressed by the chroma parameter. It is well known that color of natural matrices could be influenced by different factors, and that drying and storage conditions could determine loss of chlorophylls and/or other pigments [34,35].

**Figure 2:**
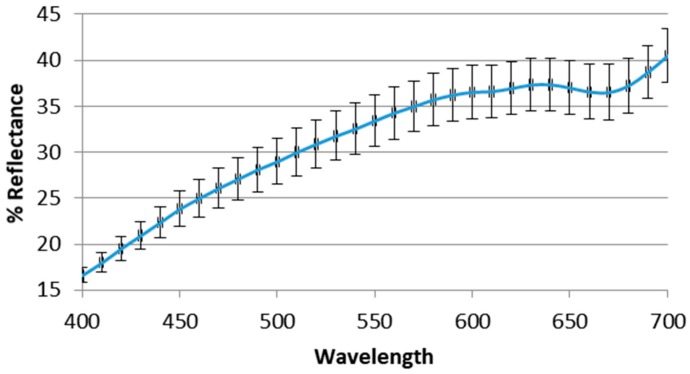
Reflectance graph of *A. mollis* aerial parts.

In fact, although chlorophylls were detected in the fresh samples, they were not yet visible in the dried ones, in term of green color. However, the total lack of information about color quality of aerial parts of plants in general, and of this matrix in particular, only allows us to give this preliminary information about colorimetric parameters and the relative reflectance curve (Figure 2).

### 6.5. Pigments Determination (Total Carotenoids, Chlorophyll a and Chlorophyll b)

**Figure 3:**
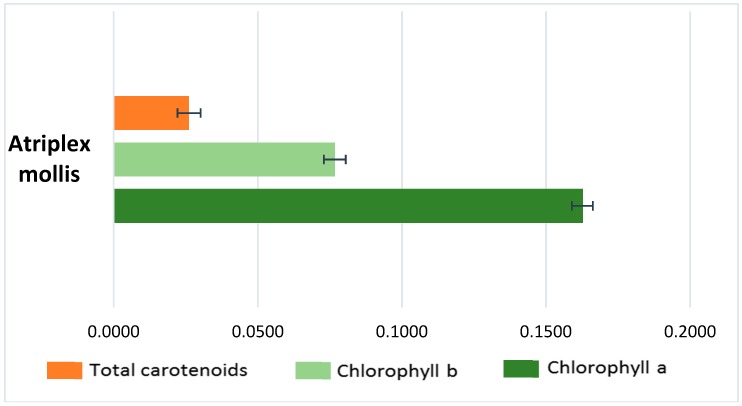
Pigment content in *A. mollis* aerial parts extract (μg/mg DW ± SD).

The amounts of total carotenoids, chlorophyll a (Chl a) and chlorophyll b (Chl b) in the organic phase of the sample from dried aerial parts are reported in Figure 3 and expressed as μg per gram of DW. The Chl a concentration proved to be double that of Chl b, as was to be expected given the importance of chlorophyll a in photosynthesis. The Chl a/b ratio value was 2.11, confirming the high content of Chl a characteristic of heliophytic species. The Chl a/b ratio is directly related not only to light but also to soil nitrogen availability [36]. Biological properties shown by chlorophylls and their derivatives consistent with cancer prevention include antioxidant and antimutagenic activity. Moreover, chlorophylls are also used in industries as food-coloring agents and are known as natural green 3 [37].

Total carotenoid content was one-sixth of that of chlorophyll a (0.0261 ± 0.004 µg/mg and 0.163 ± 0.0038 µg/mg, respectively) (Figure 3). In general, high carotenoid contents are detected for sun-exposed plants, because these molecules protect them from photo-inhibition [38].

## 7. Conclusions

This research attempted to predict for the first time the phenolics multicomponent pattern of *Atriplex mollis* aerial parts subjected to different extraction techniques and monitored by HPLC-PDA. Most of the identified and quantified compounds, including flavonoids, monoterpenes and phenolic acids, are known for their pharmacological effects on human health. Microwave-assisted extraction was shown to recover a huge number of compounds, especially rutin, *t*-ferulic acid and gallic acid, within a shorter time and without degrading the matrix up to 80 °C. Pigment analysis explored the quantitative ratios among chlorophylls (both a and b) and carotenoids. In addition, color evaluation, based on CIELAB parameters, was applied for the first time to this endemic and spontaneous Algerian plant. Further investigations may lead to isolation of other bioactive compounds followed by a rational screening of their pharmacological activity helpful for the assessment of a real nutraceutical potential.

## Figures and Tables

**Table 1:** Qualitative and quantitative phenolics profile of the main subfractions of *Atriplex mollis* aerial parts extract by maceration. In the table, the mean values and standard deviations of three independent measures are reported.

Identified Compound	Phenolics Content (µg/g DW ± SD)						
	F4 (CHCl_3_)	F7 (CHCl_3_)	F13 (CHCl_3_)	F6 (EtOAc)	F10 (EtOAc)	F16 (EtOAc)	*n*-BuOH
**Gallic acid**				0.23 ± 0.02	0.70 ± 0.07	1.5 ± 0.1	0.84 ± 0.07
**Catechin**			9.9 ± 0.9	0.94 ± 0.08	2.7 ± 0.1	34.0 ± 2.2	0.80 ± 0.06
**Chlorogenic acid**							1.0 ± 0.1
***p*-Hydroxy-benzoic acid**	0.36 ± 0.04			115 ± 8		0.49 ± 0.05	
**Vanillic acid**	0.46 ± 0.06	7.9 ± 0.7	42.2 ± 1.3	1.3 ± 0.1	0.48 ± 0.05	7.7 ± 0.7	
**Epicatechin**	2.6 ± 0.1	3.2 ± 0.2	158 ± 10	2.2 ± 0.3	4.0 ± 0.3	8.6 ± 0.8	0.31 ± 0.02
**Syringic acid**		2.2 ± 0.1	0.37 ± 0.03				
**3-Hydroxy benzoic acid**			0.19 ± 0.02				1.5 ± 0.1
**3-OH,4-MeO benzoic acid**	0.53 ± 0.05		1.9 ± 0.2	0.23 ± 0.02	6.0 ± 0.5	4.1 ± 0.4	
***p*-Coumaric acid**	24.9 ± 1.2			20.3 ± 2.3			
**Rutin**				9.1 ± 0.8	65.1 ± 5.3	1.0 ± 0.1	12.3 ± 0.2
**Sinapinic acid**			8.6 ± 0.8	1.8 ± 0.1			
***t*-** **Ferulic acid**				1.8 ± 0.2	0.93 ± 0.08		
**Naringin**	0.17 ± 0.02	5.8 ± 0.3	6.3 ± 0.2	0.17 ± 0.01		9.6 ± 0.8	
**2,3-Dimethoxy-benzoic acid**			0.28 ± 0.02				
**Benzoic acid**	2.3 ± 0.1	2.6 ± 0.3	12.6 ± 1.5	3.8 ± 0.4	0.25 ± 0.02	7.4 ± 0.5	
***o*-Coumaric acid**	0.28 ± 0.02		0.17 ± 0.01			0.84 ± 0.07	
**Quercetin**						0.96 ± 0.08	
***t*-** **Cinnamic acid**		0.51 ± 0.04					
**Naringenin**	0.16 ± 0.01	7.2 ± 0.7					0.11 ± 0.01
**Carvacrol**	0.19 ± 0.01	0.44 ± 0.02	0.28 ± 0.02				0.15 ± 0.01
**Total**	31.9	29.8	241	157.5	80.1	76.3	17.1

DW: dry weight.

**Table 2:** Qualitative and quantitative phenolics profile of *Atriplex mollis* aerial part extracts by supercritical fluid extraction.

Identified Compound	Phenolics Content (µg/g DW ± SD)	
	40 °C, 10 MPa	40 °C, 30 MPa
**Gallic acid**	0.04 ± 0.01	0.02 ± 0.01
**Catechin**	0.21 ± 0.05	0.32 ± 0.03
**Vanillic acid**	0.29 ± 0.06	1.75 ± 0.09
**Epicatechin**		0.76 ± 0.05
**Syringic acid**	0.14 ± 0.02	
**3-OH,4-MeO benzaldehyde**	0.25 ± 0.04	0.82 ± 0.03
***p*** **-Coumaric acid**	0.24 ± 0.07	0.04 ± 0.01
**Rutin**		0.06 ± 0.01
**Sinapinic acid**	0.07 ± 0.01	2.39 ± 0.08
***t-*** **Ferulic acid**	0.33 ± 0.07	0.34 ± 0.04
**Naringin**	0.38 ± 0.06	
**2,3-diMeO benzoic acid**	0.04 ± 0.01	0.003 ± 0.001
**Benzoic acid**		0.08 ± 0.01
**Harpagoside**		0.20 ± 0.05
**Naringenin**	0.06 ± 0.01	
**Total**	2.05	6.78

DW: dry weight.

**Table 3:** Qualitative and quantitative analysis of the phenolics profile of *Atriplex mollis* microwave-assisted extracts.

Identified Compound	Phenolics Content (µg/g DW ± SD)						
	40 °C, 10 min, Water	60 °C, 10 min, Water	80 °C, 10 min, Water	100 °C, 10 min, Water	120 °C, 10 min, Water	80 °C, 5 min, Water	80 °C, 15 min, Water
**Gallic acid**	59.6 ± 1.6	58.7 ± 1.8	21.9 ± 1.5	76.6 ± 5.2	63.9 ± 4.2		
**Catechin**			55.5 ± 3.8				
**Chlorogenic acid**	30.8 ± 2.4						
***p*-Hydroxy-benzoic acid**	34.7 ± 3.5	37.9 ± 1.8	19.8 ± 1.3	5.16 ± 0.09			6.18 ± 0.09
**Vanillic acid**	115 ± 12	8.62 ± 0.9	125 ± 13	16.4 ± 0.8	16.8 ± 0.9		137 ± 11
**3-OH,4-MeOH benzaldehyde**					21.8 ± 1.5		
***p*-Coumaric acid**		15.9 ± 1.1	12.7 ± 1.0	14.9 ± 1.1	16.3 ± 1.7	5.42 ± 0.08	6.23 ± 0.09
**Rutin**	377 ± 29	411 ± 32	486 ± 39	520 ± 45	521 ± 40	440 ± 34	552 ± 48
**Sinapinic acid**	23.3 ± 1.6		15.6 ± 1.0	19.5 ± 1.3	26.5 ±2.1	18.3 ± 1.8	18.9 ± 1.2
***t*-Ferulic acid**	80.8 ± 5.7	81.6 ± 4.9	95.5 ± 8.1	109 ± 14	112 ± 10	94.3 ± 8.3	107 ± 11
**Naringin**	41.9 ± 2.5						
**Benzoic acid**					27.9 ± 8.9		
**Total**	762.89	613.50	832.56	761.58	805.94	558.17	784.63

DW: dry weight.

**Table 4:** Colorimetric data of *A. mollis* aerial parts using an independent color-space device.

Color Parameters	Mean Value ± SD
L*	64.1 ± 2.2
a*	1.0 ± 0.4
b*	14.2 ± 0.5
C*_ab_	14.2 ± 0.5
*h_ab_*	85.9 ± 1.5
